# A Drastic Change in Background Luminance or Motion Degrades the Preview Benefit

**DOI:** 10.3389/fpsyg.2017.01252

**Published:** 2017-07-24

**Authors:** Takayuki Osugi, Ikuya Murakami

**Affiliations:** ^1^Department of Psychology, The University of Tokyo Tokyo, Japan; ^2^Japan Society for the Promotion of Science Tokyo, Japan

**Keywords:** visual marking, background change, preview search, preview benefit, inhibition

## Abstract

When some distractors (old items) precede some others (new items) in an inefficient visual search task, the search is restricted to new items, and yields a phenomenon termed the preview benefit. It has recently been demonstrated that, in this preview search task, the onset of repetitive changes in the background disrupts the preview benefit, whereas a single transient change in the background does not. In the present study, we explored this effect with dynamic background changes occurring in the context of realistic scenes, to examine the robustness and usefulness of visual marking. We examined whether preview benefit in a preview search task survived through task-irrelevant changes in the scene, namely a luminance change and the initiation of coherent motion, both occurring in the background. Luminance change of the background disrupted preview benefit if it was synchronized with the onset of the search display. Furthermore, although the presence of coherent background motion *per se* did not affect preview benefit, its synchronized initiation with the onset of the search display did disrupt preview benefit if the motion speed was sufficiently high. These results suggest that visual marking can be destroyed by a transient event in the scene if that event is sufficiently drastic.

## Introduction

Visual search is a fundamental visual function used frequently in everyday life. When we search for a target in a cluttered scene that cannot be processed all at once, search behavior is restricted to a sub-region or subset of objects small enough for attention to be focused on it/them all. In navigating through objects, or “search items,” to find behaviorally relevant information, the visual system uses a strategy of selecting some items over others. The present study focuses on an experience-based attentional process called *visual marking*, by which irrelevant “old” items that have already been present in the visual field are deprioritized and excluded from the search, while subsequently presented “new” items are prioritized (Watson and Humphreys, 1997). When some of the distractors are displayed in an initial display (the “preview display”) and the remaining ones including the target are added in a subsequent display (the “search display”), target detection is more efficient than when all items appear simultaneously, and the reaction time (RT) for detection is often comparable to when only new items are presented. This advantage in search efficiency is called the *preview benefit*.

It is generally believed that the preview benefit includes the facilitation of new items gained by automatic attentional capture to the onset of new items (e.g., Donk and Theeuwes, 2001) or by temporal grouping as a result of perceptual segmentation of new and old items during asynchronous presentations (e.g., Jiang et al., 2002). In addition to this facilitation of new items, the preview benefit arguably involves attentional inhibition of old items (e.g., Watson and Humphreys, 1997). It has been thought that the representations of the old items are encoded and maintained in a kind of memory template, helping the observer to exclude the old items from the subsequent search. Empirical support for this account was obtained with the probe detection procedure (Watson and Humphreys, 2000); when a probe dot was presented near one of the old items rather than new items while the preview search task was being conducted, accuracy for the probe detection became lower, but there was no difference in probe detection accuracy between the old and new items’ locations when the probe appeared in every trial. The impaired probe detection was attributed to inhibition of endogenous attention at the locations of the old items, one of the mechanisms of deprioritization by visual marking. Furthermore, recent studies have demonstrated that visual marking reduces contrast sensitivity at the previewed locations (Allen and Humphreys, 2007; Osugi and Murakami, 2014). These findings suggest that an inhibitory template for visual marking benefits visual search by diverting limited attentional resources, such as time and resolution, away from previewed locations and reserving them for the target search among new items.

Some unique characteristics of preview benefit have been pointed out in the literature (for a review, see Watson et al., 2003). For example, preview benefit can be simultaneously given to up to around 15 new items when searching for a single target (Theeuwes et al., 1998), and six or seven new items can be prioritized when all new items have to be pointed at (e.g., Watson and Kunar, 2012). The observed capacity is much greater than the estimated capacity for onset capture, which is approximately four items (Yantis and Johnson, 1990; Yantis and Jones, 1991); this great capacity is viewed as supporting evidence for the contribution of visual marking in addition to that of onset capture (Osugi et al., 2016). Furthermore, the preview benefit is abolished when old items suddenly alter their shapes during the preview period (Watson and Humphreys, 1997, 2002). In contrast, mere luminance or color changes in old items do not affect preview benefit (e.g., if old and new items are differently colored; for related findings, see also Kunar et al., 2003; Watson et al., 2008). Furthermore, preview benefit persists through shape changes of old items if semantic information pertaining to the items is retained (Osugi et al., 2010). Taken together, these lines of evidence point to a role of top-down processing in the maintenance of visual marking in the presence of disruptive, bottom-up signals (see also Osugi and Kawahara, 2012).

Two memory systems arguably play a role in prioritizing new items during search (Jiang and Wang, 2004; Emrich et al., 2008; Al-Aidroos et al., 2012; Watson and Kunar, 2012; Yamauchi et al., 2017). One is a high-capacity memory system keeping track of asynchrony between the new and old items, and the other is a limited-capacity memory system (e.g., visual working memory) retaining the locations of old items. Al-Aidroos et al. (2012) demonstrated that preview benefit was more effective when the number of previewed distractors was less than the capacity limit of the latter memory system, and that limited-capacity memory also contributed to preview benefit. Researchers have also demonstrated that high-capacity memory is easily degraded during search when observers localize and respond to new items (Watson and Kunar, 2012), execute a saccade (Emrich et al., 2008), or make a sequential shift of attention (Yamauchi et al., 2017). By contrast, limited-capacity memory can survive these changes, as suggested from some preview benefits attributable to active inhibition of old distractors (Jiang and Wang, 2004; Al-Aidroos et al., 2012).

One critical difference between a laboratory search task and a more realistic search task is whether search items are presented on a blank background or on a complex and dynamically changeable background (Wolfe et al., 2002; Neider and Zelinsky, 2006). In a situation like car driving, for example, there are many motion- and luminance-change events in the scene, requiring more complex computation to detect relevant information among them. Sometimes, background luminance changes occur without associated changes in luminance of objects within the scene; for example, when objects are backlit, backlight changes will drastically affect the light intensity of the background surrounding the objects but not that of the objects themselves. The background can be abruptly set in motion as well, as in search for static objects on a street corner in the presence of a crowd of people that start walking all together, such as when triggered by a traffic signal change. Furthermore, when we shift our gaze around natural environments, saccades not only bring new local stimuli onto the center of the visual field but also simultaneously change the background. Although it is important to assess the generalizability of laboratory-based findings on visual marking by asking how background changes might affect search performance, there have been very few studies directly addressing the issue of background changes in the preview search task. Jiang et al. (2002) reported that when the luminance of a background grid changed from low to high at the onset of new items, the change did not affect search performance; in contrast, when the same luminance change occurred in the search items rather than the background, preview benefit was degraded. From these findings, it was argued that visual marking is influenced by sudden changes occurring only in marked locations, and is insensitive to changes elsewhere.

A recent study reexamined this background-independent view by employing static and dynamic random-noise displays and manipulating a combination of background noises presented in addition to the preview and search displays (Osugi and Murakami, 2015). When the background noise was continually displaced throughout each trial or when it changed from dynamic to static noise at the onset of the search display, the preview benefit remained; in contrast, when the background was changed from static to dynamic noise at the onset of the search display, this task-irrelevant background event abolished the preview benefit. Further, at the onset of the search display, preview benefit was disrupted by 2–3 transient changes in background, but not by a single transient change, suggesting that at least two background changes are required to influence visual marking. This result is consistent with a previous finding by Watson et al. (2011), who showed that repetitive shape changes in old items are associated with stronger reductions in preview benefit.

Despite this suggestion, the investigation of this effect has to be broadened beyond the experimental situation to one where static random noise is replaced by dynamic random noise. Osugi and Murakami (2015) examined the basic phenomenon of background changes to visual marking, using dynamic random noise to manipulate the number of background changes. However, because dynamic random noise contains motion signals in all directions and at all speeds, it remains unclear what in particular in these signals is able to remove the preview benefit. Furthermore, dynamic random noise is far from the first approximation of the natural world—in real situations, sudden changes in luminance of the background region can easily occur under varying illumination conditions, and the entire scene can retinally make continuous relative motions when the eyes move to track a moving object or when the vantage point moves in the environment while maintaining fixation on a stationary object. In these situations, even though background changes or motions may usually be irrelevant to attentional selection of objects, the visual system does adopt take a strategy of complete neglect, because sometimes these signals convey important information associated with the appearance of new objects. Indeed, a recent study demonstrated that task-irrelevant but behaviorally important scene changes capture attention (Kawahara et al., 2012). Therefore, it is important to ask what type and what extent of background changes critically degrade the preview benefit. To this end, we here introduced two new types of background change: a change in overall luminance (Experiment 1) and the initiation of coherent motion (Experiment 2). We also examined whether the degree of preview benefit disruption increases with the extent of background change. Because not a single transient change but only two or more consecutive changes in background random noise were found to be intense enough to disrupt visual marking (Osugi and Murakami, 2015), we tested the generality of the principle that the visual system tolerates a background change as if it were ecologically mundane insofar that the change is not sufficiently intense, but that visual marking is deteriorated if the change exceeds a certain extent. Indeed, a change in luminance and the initiation of coherent motion both capture attention, but only when the changes are intense enough (e.g., Rauschenberger, 2003; Sunny and von Mühlenen, 2014), suggesting that a sufficiently large luminance change and the rapid initiation of coherent motion can eventually be treated as behaviorally important signals, and thus can capture attention. Similarly, visual marking may be automatically disrupted wherever a sufficient amount of change occurs.

## General Methods

### Participants

All participants had normal or corrected-to-normal visual acuity. This study was carried out in accordance with the recommendations of the Ethical Principles of the American Psychological Association; all subjects gave written informed consent in accordance with the Declaration of Helsinki. The protocol was approved by the institutional ethics committee of the Graduate School of Humanities and Sociology at the University of Tokyo.

### Stimuli and Apparatus

**FIGURE 1:**
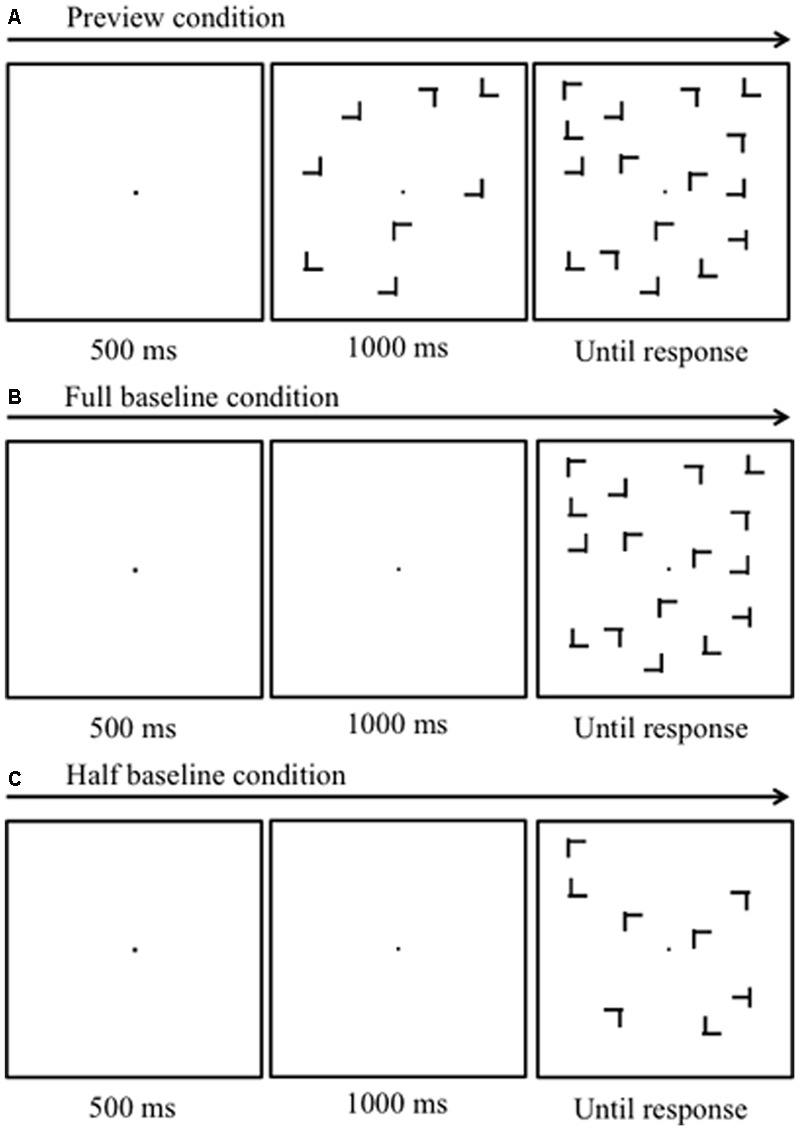
Schematic diagrams of the stimulus sequences. **(A)** Preview search condition. Half of the items appeared first, followed by the remaining items, including the target 1000 ms later. **(B)** Full-baseline condition. All items appeared simultaneously. **(C)** Half-baseline condition. Items whose number was the same as that of the new items in the preview search condition, appeared simultaneously.

**FIGURE 2:**
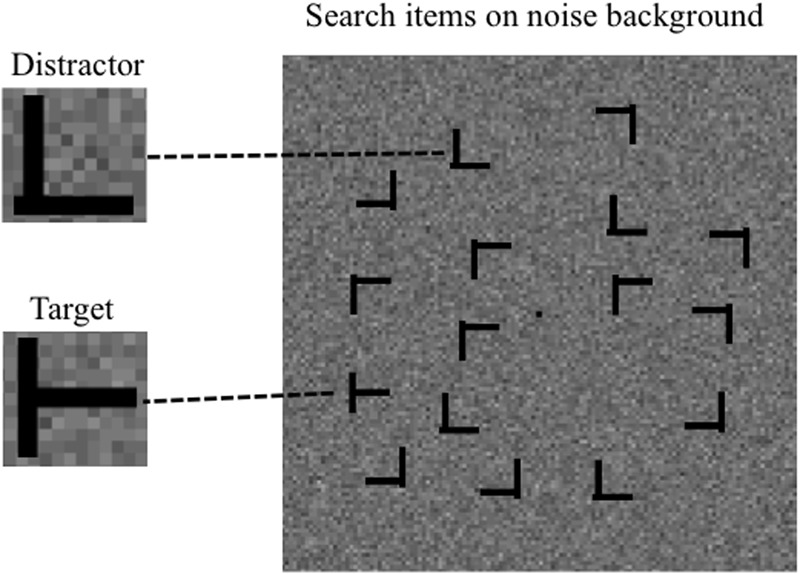
An exemplar screenshot of the search display with background noise.

The stimuli were displayed on a CRT monitor (Iiyama HM204DA, 1024 pixels × 768 pixels, refresh rate 60 Hz, mean luminance 19.62 cd/m^2^) via the stimulus processor Bits# (Cambridge Research Systems, Kent, United Kingdom), controlled by a computer (Apple Mac Pro), using Matlab and the Psychophysics Toolbox (Brainard, 1997; Pelli, 1997; Kleiner et al., 2007). The viewing distance was 57 cm. The monitor was gamma-corrected to achieve linear luminance output. A fixation dot (0.23° × 0.23°) was presented at the center of the display. Search items consisted of uppercase letters *T*s and *L*s subtending 0.94° in height and width. The width of each line segment was 0.16°. The line segments forming the *L*s had a 0.08° offset at their junctions. The target was a *T* rotated by 90° or 270°, whereas the distractors were *L*s rotated by 0°, 90°, 180°, or 270°. In all experiments, the items were presented at pseudo-randomly selected locations of an invisible 7 × 7 matrix subtending 13.14° in height and width (**Figure 1**); the target could appear at any of these locations with equal probability. In Experiment 1, the items were in medium luminance (19.62 cd/m^2^), whereas the background was in either low (<0.01 cd/m^2^) or high (47.5 cd/m^2^) luminance. In Experiment 2, the items were in low luminance (<0.01 cd/m^2^) and were presented on random noise subtending 20.02° in height and width. The noise consisted of 256 × 256 dots (2 pixels × 2 pixels each), with contrast levels sampled from a Gaussian distribution with a mean of 0% and a standard deviation of 12% around the mean luminance level of 19.62 cd/m^2^ (see **Figure 2**).

### Design and Procedure

Each experiment had a 3 × 3 design with two within-observer factors: three search types (“preview,” “full-baseline,” and “half-baseline”; **Figures 1A–C**, respectively), and three set sizes (4, 8, and 16 items). Under the “preview” condition (**Figure 1A**), a trial began with the presentation of a fixation dot for 500 ms, followed by the onset of the old items and then that of the new items, with a stimulus-onset asynchrony of 1000 ms. The first and second displays of items will be hereafter called “preview” and “search” displays, respectively. Participants had been informed that the preview display never contained the target. In the “full-baseline” condition, the fixation dot was presented alone for 1500 ms, after which all the items were displayed simultaneously. The “half-baseline” condition was identical to the “full-baseline” condition except that the number of the items was halved from the nominal set size. In each experiment, observers completed nine blocks of trials (three blocks for each search type, with the order of search types counterbalanced across observers; for each observer, all search types were tested once before being repeated, such as “ABC ABC ABC”). Each block consisted of 30 trials (10 for each set size); experimental blocks were preceded by three 30-trial practice blocks, one for each of the three search types.

Observers were asked to find a *T* and to indicate its rotation angle by pressing the “6” key to indicate 90° or the “4” key to indicate 270° on a number-pad keyboard. RT was measured. At the end of each trial, feedback was provided on the RT of target detection and the correctness of the T-orientation response (“correct” or “incorrect”). When the response was incorrect, a 1000-Hz tone was presented for 20 ms. Pressing the “5” key triggered the next trial.

### Data Analysis

For each observer, the mean RT and correct response rate were calculated for each condition. RTs for incorrect responses, those below 200 ms, and those above 6000 ms were excluded from the analysis^1^. The hallmark of the preview benefit is that the search function—RT plotted against set size—under the “preview” condition has a significantly shallower slope than that under the “full-baseline” condition. Moreover, maximal preview benefit is understood to occur when the search slope under the “preview” condition is indistinguishable from that under the “half-baseline” condition. To assess these relationships, we performed an analysis of variance (ANOVA) for RT to examine whether there was a preview benefit in each experiment. The results of the ANOVA for RT are summarized in **Table 1**, search function statistics are summarized in **Table 2**, and error rates are shown in **Table 3**. The error rate was below 4% in all cells of the factorial design (mean ± SD, 1.81 ± 0.71% in Experiment 1 and 1.98 ± 0.77% in Experiment 2); therefore, no further analysis was conducted in that regard.

**Table 1:** ANOVA results for Experiments 1–2.

	Full vs. Pre vs. Half			Full vs. Pre			Half vs. Pre		
	*F*	*p*	$\eta_{p}^{2}$	*F*	*p*	$\eta_{p}^{2}$	*F*	*p*	$\eta_{p}^{2}$
**Experiment 1**									
No change									
Task type	80.07	0.01	0.84	134.45	0.01	0.90	10.33	0.01	0.41
Set size	108.40	0.01	0.88	99.34	0.01	0.87	77.09	0.01	0.84
Task type × set size	24.96	0.01	0.62	21.78	0.01	0.59	4.72	0.02	0.24
Change									
Task type	85.95	0.01	0.85	67.86	0.01	0.82	59.64	0.01	0.80
Set size	126.89	0.01	0.89	113.4	0.01	0.88	87.14	0.01	0.85
Task type × set size	19.28	0.01	0.56	1.51	0.24	0.09	17.73	0.01	0.54
**Experiment 2**									
Low speed									
Continuous motion									
Task type	47.32	0.01	0.76	42.51	0.01	0.74	8.91	0.01	0.37
Set size	94.12	0.01	0.86	83.68	0.01	0.85	71.0	0.01	0.83
Task type × set size	17.15	0.01	0.53	16.13	0.01	0.52	3.55	0.04	0.19
Motion initiation									
Task type	31.98	0.01	0.68	19.48	0.01	0.56	17.37	0.01	0.54
Set size	77.29	0.01	0.84	71.64	0.01	0.83	95.54	0.01	0.86
Task type × set size	13.42	0.01	0.47	6.89	0.01	0.31	6.82	0.01	0.31
Middle speed									
Continuous motion									
Task type	114.08	0.01	0.88	152.02	0.01	0.91	17.56	0.01	0.54
Set size	185.19	0.01	0.93	174.59	0.01	0.92	138.51	0.01	0.90
Task type × set size	34.09	0.01	0.69	21.69	0.01	0.59	10.68	0.01	0.42
Motion initiation									
Task type	88.77	0.01	0.86	53.52	0.01	0.78	49.06	0.01	0.77
Set size	148.05	0.01	0.91	135.52	0.01	0.90	142.33	0.01	0.90
Task type × set size	23.03	0.01	0.61	7.06	0.01	0.32	20.54	0.01	0.58
High speed									
Continuous motion									
Task type	47.25	0.01	0.77	41.68	0.01	0.75	6.69	0.01	0.32
Set size	182.96	0.01	0.93	163.67	0.01	0.92	155.4	0.01	0.92
Task type × set size	17.76	0.01	0.56	7.08	0.01	0.34	15.25	0.01	0.52
Motion initiation									
Task type	66.36	0.01	0.83	33.19	0.01	0.70	33.85	0.01	0.71
Set size	178.66	0.01	0.93	195.69	0.01	0.93	110.59	0.01	0.89
Task type × set size	13.48	0.01	0.49	0.84	0.44	0.06	17.48	0.01	0.56

**Table 2:** Search function statistics for Experiments 1–2.

	Slope (ms/item)			Intercept (ms)		
	Pre	Full	Half	Pre	Full	Half
**Experiment 1**						
No change	45.7	71.4	32.7	427.8	464.0	478.1
Change	64.4	73.1	31.7	432.1	487.5	528.1
**Experiment 2**						
Low speed					
Continuous motion	37.3	57.7	28.8	459.4	454.7	473.3
Motion initiation	41.3	61.6	27.5	445.6	451.1	486.6
Middle speed					
Continuous motion	35.5	53.0	27.4	417.7	454.5	440.3
Motion initiation	42.7	55.2	26.0	393.5	456.5	455.2
High speed					
Continuous motion	46.0	56.8	30.5	494.2	599.2	560.1
Motion initiation	53.0	54.1	31.1	445.3	593.2	519.1

**Table 3:** Mean error rates in Experiments 1–2.

	Search type and set size								
	Pre			Full			Half		
	4	8	16	4	8	16	4	8	16
**Experiment 1**									
No change	1.3	2.9	1.7	1.9	0.6	1.7	1.3	2.3	1.3
Change	2.1	1.5	3.3	2.3	0.6	1.7	2.1	2.7	1.3
**Experiment 2**									
Low speed									
Continuous motion	2.3	4.0	2.7	2.5	0.6	2.1	1.5	3.8	1.9
Motion initiation	1.9	3.3	2.9	2.1	1.9	2.3	2.1	2.7	2.7
Middle speed									
Continuous motion	1.7	0.8	1.5	1.3	2.1	1.7	2.3	2.1	2.7
Motion initiation	1.3	1.9	1.5	2.5	1.9	1.9	1.3	2.3	1.7
High speed									
Continuous motion	2.5	1.5	3.5	1.5	1.2	3.2	0.7	1.8	1.5
Motion initiation	1.5	1.2	3.2	0.8	0.8	1.8	1.3	1.5	1.7

We also calculated a statistic called “preview efficiency” (PE) as an index of the strength of the preview benefit (Blagrove and Watson, 2010). PE is the difference in search slope between the “full-baseline” and “preview” conditions, divided by the difference in search slope between the “full-baseline” and “half-baseline” conditions; PE closer to 1 indicates greater preview benefit, and PE equals to 0 means no preview benefit.

To examine the contribution of inhibition based on capacity-limited working memory, a statistic called “item benefit,” which is an index of the number of inhibited distractors at each set size, was calculated as follows (Al-Aidroos et al., 2012). First, RT benefit at each set size was calculated based on the difference in RT between the preview and full-baseline conditions at that set size. Second, each RT benefit was divided by the search slope under the full-baseline condition.

## Experiment 1

### Methods

In Experiment 1, we examined whether a single luminance change in background affects the preview benefit. Fifteen adults (aged 19–32 years) who were unaware of the purpose of the study participated, along with the first author. In the “no-change” condition, we checked if preview benefit was obtained in the same way as in conventional studies of visual marking when there was no luminance change in the background. Items were presented on a high- or low-luminance background in each trial. In the “change” condition, we examined whether a single luminance change in background affected preview benefit. In each trial, a bright (or dark) background used for the preview display was changed to a dark (or bright) background at the onset of the search display. The change and no-change conditions were conducted in separate sessions run on the same day, with the order counterbalanced across observers.

To determine the preview benefit, we compared the search performance under the preview, full-baseline, and half-baseline conditions. If the luminance change in background eliminates preview benefit, the search slope under the preview condition should become the same as that observed under the full-baseline condition. In contrast, if the luminance change in background is unrelated to preview benefit, the search slope under the preview condition should be shallower than that of the full-baseline condition.

### Results

#### No-Change Condition

**FIGURE 3:**
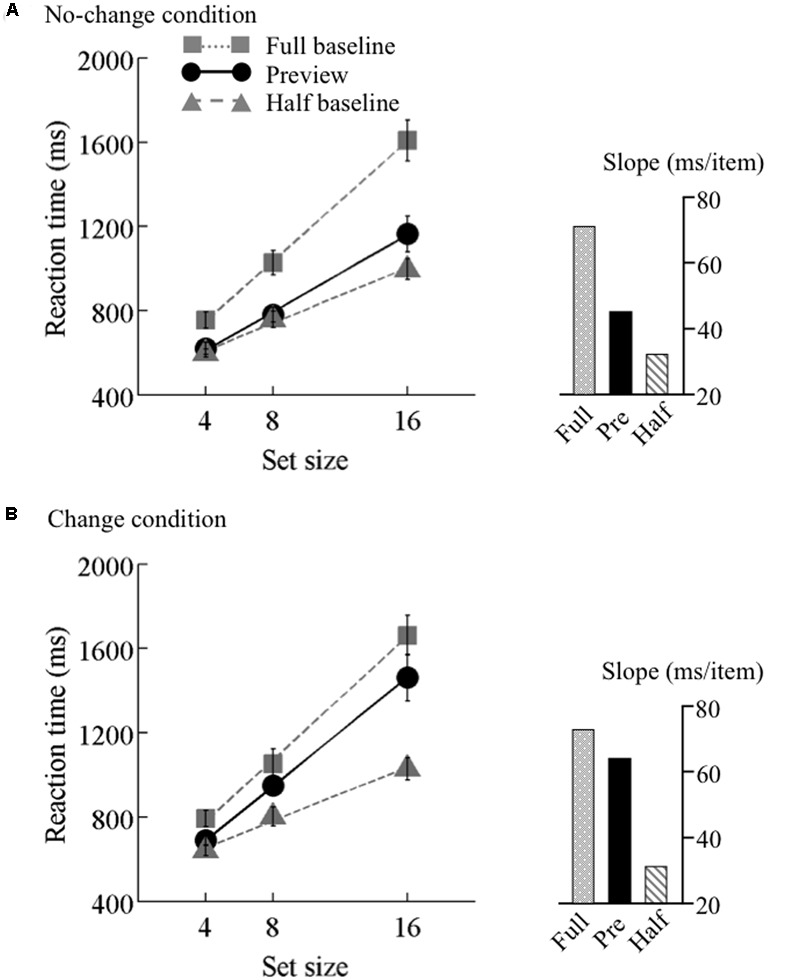
Results of Experiment 1. Mean reaction times as a function of set size and search type (the preview, full-baseline, and half-baseline conditions) under the **(A)** no-change and **(B)** change conditions. Error bars represent standard errors. Each inset shows the slopes of the linear regression.

The search function under the no-change condition is plotted in **Figure 3A**. There were two levels of background luminance, but the RT data were merged because no systematic difference was observed between these luminance conditions. PE calculated from the mean RT data was 0.67. A 3 × 3 ANOVA for RT with search type (preview, full-baseline, and half-baseline) and set size (4, 8, and 16) as within-observer factors revealed significant main effects of search type (*F*_2,30_ = 80.07) and set size (*F*_2,30_ = 108.4) and also a significant interaction (*F*_4,60_ = 24.96), indicating that the search slopes were different among the three search types. Thus, we next performed two separate two-way within-observer ANOVAs, as are commonly implemented in visual marking studies, for detailed comparison between the search types (Watson and Humphreys, 1997). When the RT data were compared between the preview and full-baseline conditions, the interaction between search type and set size was significant (*F*_2,30_ = 21.78); when the RT data were compared between the preview and half-baseline conditions, the interaction was also significant (*F*_2,30_ = 4.72). Therefore, there was a preview benefit, though submaximal, when the items were presented on a bright or dark background.

#### Change Condition

The search function under the change condition is plotted in **Figure 3B**. PE calculated based on the mean RT data was 0.21. As in the no-change condition, a 3 × 3 ANOVA revealed significant main effects of search type (*F*_2,30_ = 85.96) and set size (*F*_2,30_ = 126.89), and also a significant interaction (*F*_4,60_ = 19.28). In a separate ANOVA to compare the data between the preview and full-baseline conditions, however, the interaction was *not* significant (*F*_2,30_ = 1.51); but in contrast, the interaction was significant in the comparison between the preview and half-baseline conditions (*F*_2,30_ = 17.73). Therefore, the slope under the preview condition (64.4 ms/item) did not significantly differ from that under the full-baseline condition (73.1 ms/item), but did differ from that under the half-baseline condition (31.7 ms/item). These results indicate that a single transient change in background luminance abolished the preview benefit, and thus that such a change is sufficient for disrupting visual marking as long as the change is drastic.

In this experiment, there were two types of background change, namely luminance increment and decrement. To determine possible differences in search performance between these two types, we conducted ANOVAs separately for these conditions and found that the interaction between the preview and full-baseline conditions was non-significant for both the luminance increment and decrement conditions (*F*_2,30_ = 1.06, *p* = 0.36 for the increment, *F*_2,30_ = 1.08, *p* = 0.35 for the decrement), indicating that increment and decrement equally removed preview benefit.

It is possible that some of the old items were correctly ignored but others failed to be ignored and affected the search for the target. That is, the transient luminance change may have dampened inhibition of some items but not others, contributing to the overall RT difference between the preview and full-baseline conditions. To explore this possibility, we examined whether the item benefit (Al-Aidroos et al., 2012) increased with set size (**Table 4**). First of all, item benefit was significantly above zero in all conditions [*t*s(15) > 2.9, *p*s ≤ 0.01]. Under the no-change condition, ANOVA for item benefit with set size (4, 8, and 16) as a factor revealed a significant main effect (*F*_2,30_ = 24.94, *p* < 0.001). Then, multiple comparisons by Ryan’s method confirmed that item benefit significantly differed between set sizes 4 and 8, between 4 and 16, and between 8 and 16 [*t*s(30) > 2.44, *p*s< 0.05], that is, item benefit increased with set size, a hallmark of visual marking. Under the change condition, however, the main effect of set size was not significant (*F*_2,30_ = 1.6, *p* = 0.21). Therefore, given the background luminance change, visual marking was lost, but a constant small number (perhaps one or two) of the old items could still be ignored with the help of some capacity-limited system such as visual working memory.

**Table 4:** Item benefits in Experiments 1–2.

	Item benefit		
	4	8	16
**Experiment 1**			
No change	2.0	3.5	6.3
Change	1.4	1.4	2.8
**Experiment 2**			
Low speed			
Continuous motion	1.3	3.0	5.1
Motion initiation	1.4	2.4	3.8
Middle speed			
Continuous motion	2.0	3.4	6.0
Motion initiation	1.7	3.9	4.2
High speed			
Continuous motion	2.7	4.3	4.8
Motion initiation	2.2	3.5	2.4

## Experiment 2

### Methods

In Experiment 2, we examined the generality of the influence of background change on the preview search by introducing another type of background change (namely, initiation of coherent motion). In total, 46 adults (aged 19–33 years) participated. Under the “motion initiation” condition, static background noise was presented during the fixation and preview displays; search items were superimposed on the static background noise in the preview display, and the noise pattern started to move or translate in one of the four directions (up, down, left, or right) at the onset of the search display. Under the “continuous motion” condition, a coherently translating background noise moving in one of the four directions was presented throughout the fixation, preview, and search displays. Half of the observers completed the block of the motion initiation condition prior to the block of the continuous motion condition, whereas the others completed the two blocks in reverse order.

We also manipulated the speed of the background motion (6.25, 12.5, and 25°/s); for each speed, there were 16 observers (15 adults, who were newly recruited and were unaware of the purpose of the study, and the first author). The same noise pattern was shifted every 100 ms in the 6.25 and 12.5°/s speed conditions, whereas the noise was shifted every 50 ms in the 25°/s speed condition.^2^ Previous studies demonstrated that attentional capture to motion onset depends on the initial abrupt displacement of moving items (Sunny and von Mühlenen, 2014). Thus, if the speed of the background motion is sufficiently high, such a change may capture attention and thereby attenuate the effect of visual marking.

### Results

**FIGURE 4:**
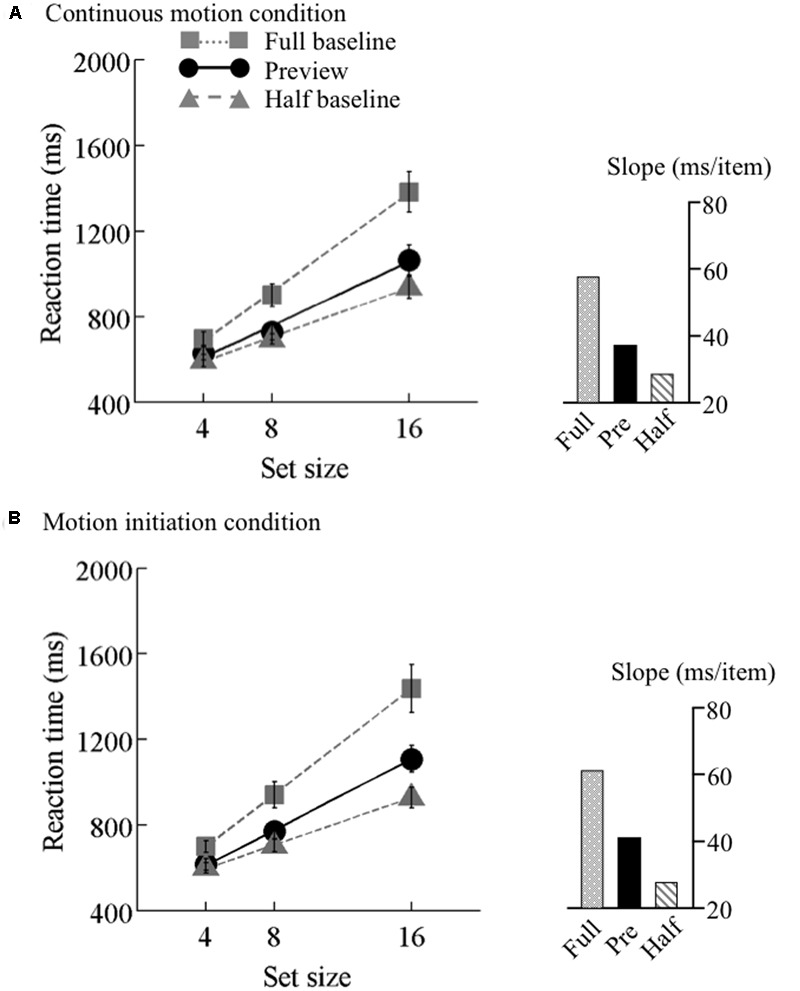
Results of Experiment 2, low speed. Mean reaction times as a function of set size and search type (the preview, full-baseline, and half-baseline conditions) for the low speed (6.25°/s) under the **(A)** continuous motion and **(B)** motion initiation conditions. Other conventions are identical to those of **Figure 3**.

**FIGURE 5:**
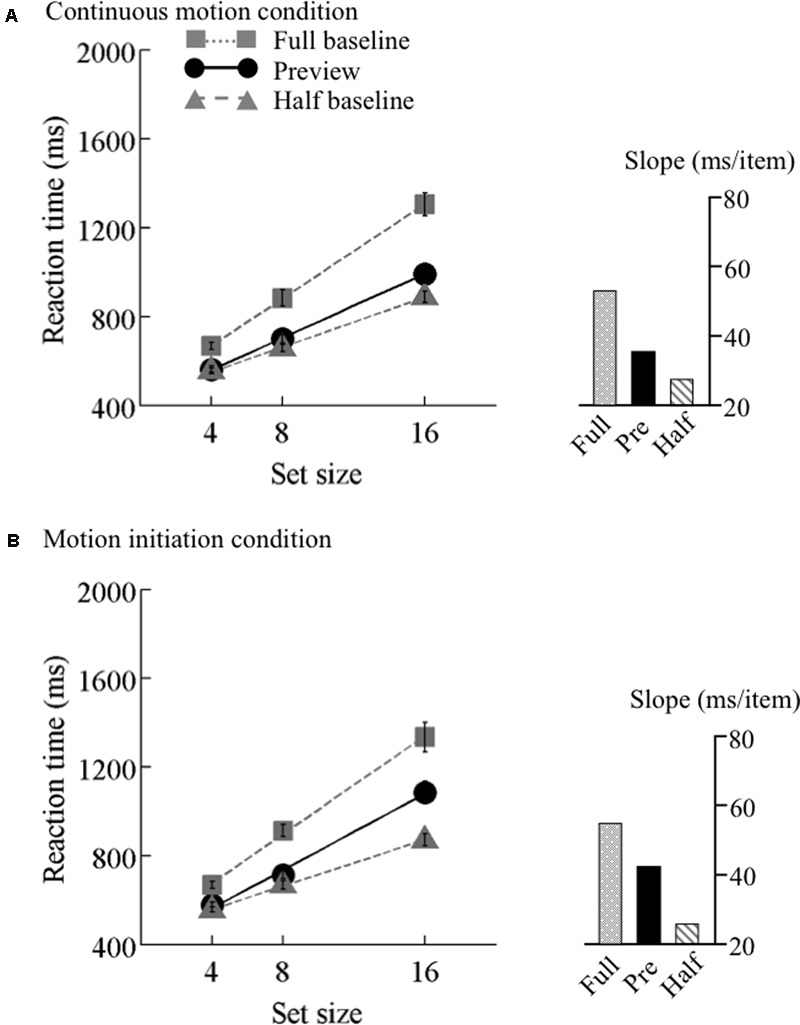
Results of Experiment 2, middle speed. Mean reaction times as a function of set size and search type (the preview, full-baseline, and half-baseline conditions) for the medium speed (12.5°/s) under the **(A)** continuous motion and **(B)** motion initiation conditions. The conventions are identical to those of **Figure 4**.

**FIGURE 6:**
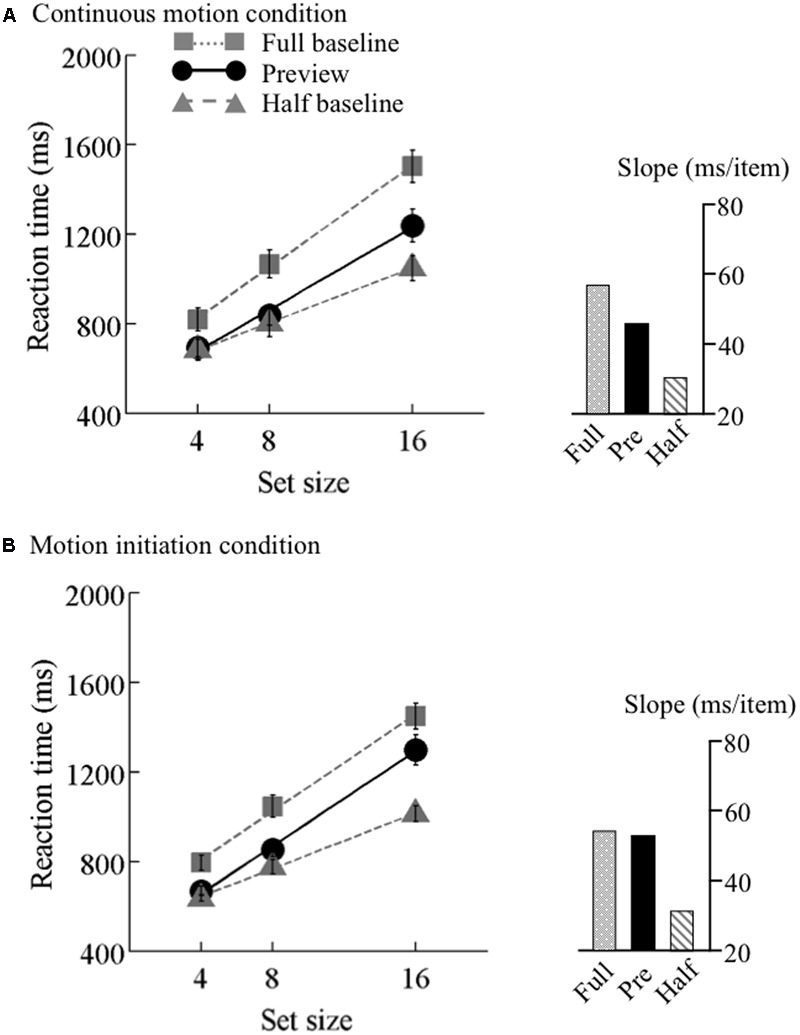
Results of Experiment 2, high speed. Mean reaction times as a function of set size and search type (the preview, full-baseline, and half-baseline conditions) for the high speed (25 °/s) under the **(A)** continuous motion and **(B)** motion initiation conditions. The conventions are identical to those of **Figure 4**.

The search functions for Experiments 2 are plotted in **Figures 4–6**. Under the continuous motion condition, PEs calculated from the mean RT data were 0.71 at 6.25°/s, 0.68 at 12.5°/s, and 0.41 at 25°/s. Under the motion initiation condition, PEs were 0.60 at 6.25°/s, 0.43 at 12.5°/s, and 0.05 at 25°/s. At 25°/s, one observer was excluded from the analysis because his error rate under the motion initiation condition was above 17%.

### Continuous Motion Condition

A mixed-model ANOVA with search type (preview and full-baseline) and set size (4, 8, and 16) as within-observer factors and motion speed (6.25, 12.5, and 25°/s) as a between-observer factor revealed significant main effects of search type (*F*_1,44_ = 170.48, *p* < 0.001), set size (*F*_2,88_ = 374.14, *p* < 0.001), and motion speed (*F*_2,44_ = 3.81, *p* = 0.03). The interaction between search type and set size was significant (*F*_2,88_ = 39.86, *p* < 0.001). However, the interactions between search type and motion speed (*F*_2,44_ = 0.15, *p* = 0.86) and between set size and motion speed (*F*_4,88_ = 0.81, *p* = 0.52), and the three-way interaction (*F*_4,88_ = 1.54, *p* = 0.20) were non-significant, indicating that overall RT, search slope, and preview benefit did not differ across the three motion speeds.

We also tested the effect of motion speed separately. 3 × 3 ANOVAs with search type (preview, full-baseline, and half-baseline) and set size (4, 8, and 16) as within-observer factors were performed separately for the three speeds, revealing significant interactions (*F*_4,60_ = 17.15 at 6.25°/s, *F*_4,60_ = 34.09 at 12.5°/s, and *F*_4,56_ = 17.76 at 25°/s). At all the speeds, a significant interaction was identified between the preview and full-baseline conditions (*F*_2,30_ = 16.13 at 6.25°/s, *F*_2,30_ = 21.69 at 12.5°/s, and *F*_2,28_ = 7.08 at 25°/s), and another between the preview and half-baseline conditions (*F*_2,30_ = 3.55 at 6.25°/s, *F*_2,30_ = 10.68 at 12.5°/s, and *F*_2,28_ = 15.25 at 25°/s). That is, preview benefit was present, though submaximal, at all the speeds in the presence of continuous coherent motion of the background (such as throughout a trial).

As in Experiment 1, item benefit was calculated (**Table 4**). It was significantly above zero in all conditions [*t*s(14–15) > 2.58, *p* ≤ 0.02]. A one-way ANOVA with set size as a factor was calculated for each of the three speeds. A significant main effect of set size was identified for each speed (*F*_2,30_ = 13.55, *p* < 0.001 at 6.25°/s, *F*_2,30_ = 25.73, *p* < 0.001 at 12.5°/s, and *F*_2,28_ = 3.85, *p* = 0.03 at 25°/s). Multiple comparisons indicated that, as predicted from visual marking, item benefit significantly differed between set sizes 4 and 8, between 4 and 16, and between 8 and 16 for the 6.25°/s condition [*t*s(30) > 2.36, *ps* < 0.05] and the 12.5°/s condition [*t*s(30) > 2.45, *ps* < 0.05], whereas a significant difference was found only between 4 and 16 for the 25°/s condition [*t*(28) = 2.63, *p* < 0.05].

### Motion Initiation Condition

A mixed-model ANOVA with search type (preview and full-baseline) and set size (4, 8, and 16) as within-observer factors and motion speed (6.25, 12.5, and 25°/s) as a between-observer factor revealed significant main effects of search type (*F*_1,44_ = 84.38, *p* < 0.001) and set size (*F*_2,88_ = 334.09, *p* < 0.001). The main effect of motion speed was marginally significant (*F*_1,44_ = 2.99, *p* = 0.06). The interaction between search type and set size was significant (*F*_2,88_ = 10.14, *p* < 0.001), but the interactions between search type and motion speed (*F*_2,44_ = 0.31, *p* = 0.73) and between set size and motion speed (*F*_4,88_ = 0.23, *p* = 0.92) were non-significant. Furthermore, a marginally significant three-way interaction (*F*_4,88_ = 2.44, *p* = 0.05), and the simple interactions between search type and set size were significant at 6.25°/s (*F*_2,88_ = 9.89, *p* < 0.001) and 12.5°/s (*F*_2,88_ = 4.34, *p* = 0.02), but not at 25°/s (*F*_2,88_ = 0.78, *p* = 0.46); taken all together, these findings indicate that the preview benefit was disrupted at this speed only.

We also tested the effect of motion speed separately. 3 × 3 ANOVAs were performed for the three speeds, revealing significant interactions (*F*_4,60_ = 13.42 at 6.25°/s, *F*_4,60_ = 23.03 at 12.5°/s, and *F*_4,56_ = 13.48 at 25°/s). At speeds of both 6.25 and 12.5°/s, significant interactions were identified between the preview and full-baseline conditions (*F*_2,30_ = 6.89 at 6.25°/s and *F*_2,30_ = 7.06 at 12.5°/s) and between the preview and half-baseline conditions (*F*_2,30_ = 6.82 at 6.25°/s and *F*_2,30_ = 20.54 at 12.5°/s). That is, a submaximal preview benefit survived the initiation of coherent motion in the background noise for these conditions. At 25°/s, however, the interaction between the preview and full-baseline conditions was not significant (*F*_2,28_ = 0.84), whereas the interaction between the preview and half-baseline conditions was significant (*F*_2,28_ = 17.48). Therefore, the initiation of background coherent motion at this speed disrupted the preview benefit.

At 6.25 and 12.5°/s, the search slope under the preview condition was shallower than that under the full-baseline condition no matter whether the background motion was continuous or initiated. Thus, background motion at these speeds did not spoil visual marking. At 25°/s, however, the search slopes indicate disruption of visual marking due to the fast background change that started synchronously with the onset of the search display. Because a conventional preview benefit occurred in the presence of continuous background motion at all speeds, impoverished item visibility *per se*, which may have been introduced by fast background motion, cannot explain the disappearance of the preview benefit at the initiation of a high-speed motion. This finding can be viewed as psychophysical evidence for the robustness and usefulness of visual marking occurring in the context of continuous background motion, which is ubiquitous in more realistic scenes than those on a computer display.

In Experiment 2, there were four directions of background motion. Is some direction of motion more disruptive than the others? In everyday life, people may be more likely to encounter a particular directional axis, perhaps left/right more likely than up/down, and so we asked whether horizontal vs. vertical motions made any difference. For the continuous motion condition, we performed ANOVA separately for the horizontal and vertical directions and found that the interaction between the preview and full-baseline conditions was significant or marginally significant at all speeds for both the horizontal (*F*_2,30_ = 5.39, *p* = 0.01 at 6.25°/s, *F*_2,30_ = 9.04, *p* < 0.001 at 12.5°/s, and *F*_2,28_ = 4.71, *p* = 0.02 at 25°/s) and the vertical (*F*_2,30_ = 11.83, *p* < 0.001 at 6.25°/s, *F*_2,30_ = 17.7, *p* < 0.001 at 12.5°/s, and *F*_2,28_ = 3.08, *p* = 0.06 at 25°/s), indicating that the same preview benefit functions irrespective of motion direction. In contrast, an interesting anisotropy emerged as we conducted ANOVA in the same way for the motion initiation condition. For the horizontal, there was a significant interaction between the preview and full-baseline conditions at 6.25 and 12.5°/s (*F*_2,30_ = 8.08, *p* = 0.002 at 6.25°/s and *F*_2,30_ = 8.05, *p* = 0.002 at 12.5°/s) but not at 25°/s (*F*_2,28_ = 0.25, *p* = 0.78). For the vertical, however, interactions between the preview and the full-baseline conditions were insignificant at all speeds (*F*_2,30_ = 1.17, *p* = 0.32 at 6.25°/s, *F*_2,30_ = 2.32, *p* = 0.12 at 12.5°/s, and *F*_2,28_ = 1.39, *p* = 0.27 at 25°/s). These results suggest that the up/down motion directions is more disruptive than the left/right directions, possibly consistent with the hypothesis that attentional mechanisms are more susceptible to uncommon perceptual events (if it is the case that vertical motions are encountered with lower probability than horizontal motions in everyday life).

Item benefit was significantly above zero in all conditions [*t*s(14–15) > 2.27, *p* ≤ 0.04] (**Table 4**). In ANOVA, the main effect of set size was marginally significant at 6.25°/s (*F*_2,30_ = 2.83, *p* = 0.07) and significant at 12.5°/s (*F*_2,30_ = 6.14, *p* < 0.001), but not at 25°/s (*F*_2,28_ = 0.98, *p* = 0.38). Multiple comparisons for the 6.25°/s condition confirmed a significant difference only between set sizes 4 and 16 [*t*(30) = 2.38, *p* < 0.05]; the same analysis for the 12.5°/s condition confirmed that the item benefit significantly differed between set sizes 4 and 8 and between 4 and 16 [*t*s(30) > 2.81, *ps* < 0.05], but not between 8 and 16 [*t*(30) = 0.41, *p* > 0.05]. This pattern of results recapitulates the disruption of visual marking and offers a possibility of a constant small number of old items still receiving active inhibition with a capacity-limited system.

## Discussion

The present study examined the generality of the principle that a drastic rather than slight change in background can deteriorate visual marking, focusing upon overall luminance change and motion initiation. In a previous study, the onset of task-irrelevant repetitive changes in the background was found to visual marking (Osugi and Murakami, 2015), but this effect may apply only to the case of static noise replaced by dynamic noise. As Experiment 1 demonstrated, the preview benefit as an index of visual marking was abolished when background luminance changed at the onset of the search display and when coherent motion at a high speed (25°/s) was started at the onset of the search display. In contrast, the preview benefit was not affected by the continuous motion itself. These findings suggest that the survival of visual marking depends on how drastic a background change is; a sufficiently drastic background change can immediately nullify visual marking, but the influence of minor changes in background such as the dynamic noise used in Osugi and Murakami (2015) may lead more gradually to an increasing number of changes and a different situation.

The present findings are related to the debate regarding whether the mechanism of visual marking monitors only marked locations and sudden changes therein. Jiang et al. (2002) demonstrated that a transient change in old items disrupted preview benefit but that background change (i.e., a change in the luminance of the background grid) did not. However, the present results indicate that the survival of the preview benefit depends on the intensity of background change. In the literature of attentional capture, a change in luminance as well as the initiation of coherent motion can capture attention only when the change is sufficiently intense (e.g., Rauschenberger, 2003; Sunny and von Mühlenen, 2014). Researchers argue that the visual system differentiates a sufficiently drastic change from other, minor changes in the scene and treats it as a behaviorally relevant signal to which attention is automatically captured (Franconeri and Simons, 2003; Forster and Lavie, 2007; Kawahara et al., 2012). In a similar way, the system responsible for visual marking may be monitoring background information in order to destroy the memory template corresponding to old distractor positions when it detects a sufficiently drastic background change to which a good amount of attentional resource should be newly allocated for urgent scrutiny. In contrast, when background change is transient or small, no change needs to occur in attentional resource allocation, because such a change would be ecologically mundane and uninteresting.

A previous study has demonstrated that repetitive shape changes in old items are associated with stronger reductions in preview benefit (Watson et al., 2011). Similarly, it has been observed that repetitive background changes are more disruptive compared with single transient changes (Osugi and Murakami, 2015), and when transient background changes occur twice or three times, preview benefit is fully abolished. One possible explanation of the gradual reduction in preview benefit is that the visual system monitors the total amount of change in the scene. In this case, the impact of minor changes would accumulate and lead to cumulative activation up to a threshold above which such activation will start affecting visual marking. As shown in the present study, when a sufficiently drastic background change occurs in a scene, a single change will be sufficient to disrupt visual marking. Therefore, repetitive changes and single changes may be explained using the same theoretical framework.

The preview benefit remained when the background was continuously and coherently moved throughout the trial. This finding is consistent with those of Osugi and Murakami’s (2015) study: preview benefit remains if there is a sufficiently long interval between the onset of repetitive changes and the onset of new items. That is, visual marking can persist even in complex, dynamically changeable environments if background change is not synchronized with the onset of new items. One reason for this may be that the visual system can filter out continuous repetitive changes as though they were distracting, irrelevant information. Using a change detection task, Becker and Vera (2007) demonstrated that repeated irrelevant changes were less disruptive to change detection for a target object if they occurred prior to the target change, indicating that they were being filtered out as distracting irrelevant information. A similar principle may play a role in preventing attention from being directed to irrelevant background motion that has already been present before the marking process is started for old distractors. Therefore, if there is sufficient time between the onset of motion and the appearance of new items, observers can successfully create a memory template for visual marking after distracting, irrelevant information in the environment is successfully filtered out.

The observed preview benefit was submaximal even when search items were presented on a blank background without any change. This is consistent with a recent demonstration in which preview benefit depends on the configuration of search items on a blank background, being maximal in a circular array but compromised in a matrix such as the one we used, possibly due to the relationship between stimulus configuration and one’s attentional window size (Osugi et al., 2016). Dependence of onset capture on attentional window size has been demonstrated in previous studies (Yantis and Johnson, 1990; Theeuwes, 1994; Belopolsky and Theeuwes, 2010), some of which have suggested a circular array as an ideal configuration for covering all items within the window. Some of the new items may fall outside it and fail to capture attention in a more complex configuration, resulting in a submaximal preview benefit. A color difference between old and new items may also be an important cue because, if their colors differ, preview benefit can remain maximal even in a complex configuration, possibly due to feature-based grouping and segmentation (Braithwaite et al., 2005; Watson et al., 2008). Further studies are needed to clarify all these issues.

In the motion initiation condition, the intercept of the search function for the preview condition was lower than for the full-baseline condition even at 25°/s (see **Figure 6**). As this result is consistent with Osugi and Murakami (2015)’s study, we argue that the onset of old items 1000 ms prior to the onset of new items provided an additional set-size-independent temporal cue for the arrival of the new items. Therefore, participants could start searching for the target more quickly under the preview condition, resulting in a decrease in intercept. Previewing old items may also work as an additional cue to help discriminate new items from background, and such a discrimination process may occur before a search process is started, only the intercept only (Wolfe et al., 2002).

The present results demonstrated that some drastic changes in background are able to degrade the preview benefit. Further, however, it is possible that not only background change itself but also apparent change in old items associated with the background change affected the preview benefit. A number of psychophysical studies have demonstrated phenomena of simultaneous contrast in which background or surrounding information can modulate the perceived brightness, motion, etc. of central stimuli (e.g., Takemura and Murakami, 2010; Kaneko and Murakami, 2012). Thus, an increase or decrease in background luminance with no change in item luminance might make the items appear darker or brighter. Similarly, the disruption of visual marking at 25°/sec might have occurred because the background motion induced apparent motion or position shift in search items. As in previous studies which demonstrated that change in luminance of old items (e.g., Watson et al., 2008) and change in position of old items (e.g., Watson and Humphreys, 1998) can modulate the preview benefit, such apparent changes in the visual properties of old items may also affect the preview benefit; further study is required to clarify this issue. For example, it is interesting to see an interobserver correlation between the preview benefit and the perceived speed of illusory motion induced to search items by background motion. The current stimulus setup is not optimal in this research direction, however, because we failed to observe any clear motion induction in the tested search items.

As noted in the Introduction, prioritizing new items during the search may involve two memory systems (Jiang and Wang, 2004; Emrich et al., 2008; Al-Aidroos et al., 2012; Watson and Kunar, 2012; Yamauchi et al., 2017): a high-capacity memory system keeping track of asynchrony between new and old items and a limited-capacity working memory system retaining old item locations (and little else on the old items). The present findings could be explained in terms of dual systems, by considering that transient change and motion onset are serving as a mask that disrupts the high-capacity memory preserving the spatial configuration of old items while leaving the working memory–based inhibition intact. In the change condition in Experiment 1 and the motion initiation condition at 25°/s, the item benefit analysis suggests that only one or two old items could still have been ignored irrespective of set size. Presumably, then, not only a high-capacity memory system but also a limited-capacity one, which would still be normally capable of retaining more than two locations of old items, should be affected by background changes, but the latter system might still exert part of its functionality despite the changes, considering the calculated item benefit constantly greater than zero.

That said, we do not know for sure whether such inhibition actually occurred. Because item benefit is calculated based on RT difference between the preview and full-baseline conditions, any factors that somehow affect RT while having nothing to do with preview also affect item benefit. As discussed above, the onset of old items provided a temporal cue for the arrival of the new items, and if participants could start searching for the target more quickly under the preview condition than under the full-baseline condition, item benefit will be overestimated. Further investigation should be required to estimate the actual number of items incurring inhibition.

In conclusion, our results demonstrate that task-irrelevant events in the background can degrade visual marking when there is a sufficiently drastic change in the background. The visual system determines whether the top-down visual marking process should be maintained by evaluating the intensity of change in the scene. A drastic change is treated as a potentially interesting/threatening event and requires reevaluation of resource allocation between the marked object locations and background information. In contrast, a minor or continuous change started prior to the activation of the marking process can be successfully ignored.

## Author Notes

(1) We also conducted an analysis with a more conservative RT exclusion procedure (a criterion of three SDs of the mean for exclusion criteria), and found the same pattern of ANOVA results. Therefore, our method for excluding RT outliers should be sufficient to catch all potential outliers.
(2) The background update interval was set at 100 ms to maintain consistency with our previous study, in which we used dynamic noise instead of coherent motion (Osugi and Murakami, 2015). At 25°/s, however, we had to change the interval from 100 to 50 ms to circumvent the issue of the maximum displacement allowed to perceive coherent motion (i.e., *d*_max_), because when the noise pattern was shifted by 2.5°, every 100 ms in the 25°/s speed condition, perception of coherent motion was lost. A Similar manipulation has been proven useful in an investigation of attentional capture to initial displacement of motion (Sunny and von Mühlenen, 2014).

## Author Contributions

TO planned the experimental design, conducted the experiment, analyzed the data, developed computer programs for the experiment, and wrote the manuscript. IM supervised the research, contributed to the interpretations of the data, and wrote the manuscript.

## Conflict of Interest Statement

The authors declare that the research was conducted in the absence of any commercial or financial relationships that could be construed as a potential conflict of interest.
